# Serotonin enhances solitariness in phase transition of the migratory locust

**DOI:** 10.3389/fnbeh.2013.00129

**Published:** 2013-10-07

**Authors:** Xiaojiao Guo, Zongyuan Ma, Le Kang

**Affiliations:** ^1^State Key Laboratory of Integrated Management of Pest Insects and Rodents, Institute of Zoology, Chinese Academy of SciencesBeijing, China; ^2^Beijing Institutes of Life Sciences, Chinese Academy of SciencesBeijing, China

**Keywords:** neurotransmitter, serotonin receptors, RNA interference, phenotypic plasticity, gregariousness, isolation, *Locusta migratoria*, behavior

## Abstract

The behavioral plasticity of locusts is a striking trait presented during the reversible phase transition between solitary and gregarious individuals. However, the results of serotonin as a neurotransmitter from the migratory locust *Locusta migratoria* in phase transition showed an alternative profile compared to the results from the desert locust *Schistocerca gregaria*. In this study, we investigated the roles of serotonin in the brain during the phase change of the migratory locust. During the isolation of gregarious nymphs, the concentration of serotonin in the brain increased significantly, whereas serotonin receptors (i.e., *5*-*HT*_*1*_, *5*-*HT*_*2*_, and *5*-*HT*_*7*_) we identified here showed invariable expression patterns. Pharmacological intervention showed that serotonin injection in the brain of gregarious nymphs did not induced the behavioral change toward solitariness, but injection of this chemical in isolated gregarious nymphs accelerated the behavioral change from gregarious to solitary phase. During the crowding of solitary nymphs, the concentration of serotonin in the brain remained unchanged, whereas *5*-*HT*_*2*_ increased after 1 h of crowding and maintained stable expression level thereafter. Activation of serotonin-5-HT_2_ signaling with a pharmaceutical agonist inhibited the gregariousness of solitary nymphs in crowding treatment. These results indicate that the fluctuations of serotonin content and *5*-*HT*_*2*_ expression are results of locust phase change. Overall, this study demonstrates that serotonin enhances the solitariness of the gregarious locusts. Serotonin may regulate the withdrawal-like behavioral pattern displayed during locust phase change and this mechanism is conserved in different locust species.

## Introduction

Phase polyphenism in locusts, a striking example of phenotypic plasticity, is important in evolution and adaptation (West-Eberhard, 2003; Pener and Simpson, 2009; Moczek, 2010). The migratory locust (*Locusta migratoria*) exhibits solitary and gregarious phases with phase-specific behavioral characteristics. Solitary individuals are inactive and exhibit repulsive behavior, whereas gregarious ones are active, show upright posture, and attract each other (Uvarov, 1966; Pener and Simpson, 2009; Burrows et al., 2011). Previous studies have shown that the patchy distribution of food resources concentrates locusts on the small scale, and the increase in population density forces solitary individuals to contact with each other and causes gregarization (Collett et al., 1998; Despland et al., 2000). The change in population density results in the reversible phase transition between solitary and gregarious phases (Despland, 2001; Guo et al., 2011). This behavioral pattern makes locusts aggregate with one another to form enormous swarms that result in severe economic and agriculture losses (Tanaka and Zhu, 2005; Brader et al., 2006). However, the mechanisms in controlling the formation of locust aggregation through new behavioral regulators are not fully characterized.

Great progresses have been made in revealing the genetic and metabolic mechanisms underlying the regulation of behavioral phase changes (Kang et al., 2004; Wu et al., 2012). Many neurotransmitters have been documented to play important roles in regulating behavioral phase changes in locusts (Anstey et al., 2009; Chen et al., 2010; Ma et al., 2011). Dopamine has been proved to regulate the gregariousness of solitary nymphs in the migratory locust (Ma et al., 2011). Our functional insights into the role of serotonin arose through the recent contradictory discovery of this chemical as a regulator of phase change in the migratory locust and desert locust (*Schistocerca gregaria*). In the migratory locust, unlike the clear function of dopamine in inducing and maintaining gregarious behavior (Ma et al., 2011), we found that the injection of serotonin in solitary nymphs partially induced gregarious-like behavior. However, long-term injection of serotonin precursor 5-Hydroxytryptophan (5-HTP) and exposure to crowding inhibited performance of gregarious behavior (Ma et al., 2011). In the desert locust, serotonin has been found to be necessary and sufficient to induce gregarious behavior (Anstey et al., 2009), but no genetic and molecular evidence has been reported. Moreover, another study shows that serotonin is not involved in attraction/avoidance behavior, which is one of the most important behavioral traits in aggregation (Tanaka and Nishide, 2012). Collectively, the results regarding the role of serotonin in regulating phase change from different locust species and different laboratories are contradictory.

When crowded with other locusts, solitary individuals are subjected to visual, olfactory and mechanosensory stimuli from their gregarious conspecifics and shift their behavior toward the gregarious phase. The brain and thoracic ganglia in the central nervous system signal olfactory/visual and mechanosensory stimuli, respectively (Simpson et al., 2001; Rogers et al., 2003; Guo et al., 2011). In the desert locust, the confirmation of serotonin function in phase change focused on the tissue of thoracic ganglia (Anstey et al., 2009). Although the insect thoracic ganglia acts as the central pattern generator to regulate the locomotion circadian rhythms and mechanosensory stimuli (Berkowitz and Laurent, 1996), the ability of the thoracic ganglia to monitor movement and other activities depends on the instruction of the brain through descending pathways (Delcomyn, 1999; Schaefer and Ritzmann, 2001). Therefore, knowing how serotonin in the brain modulates behavioral transition of migratory locusts is necessary for us to comprehensively understand the role of serotonin in the phase change of locusts.

In this study, we measured serotonin levels in the brain over a time course of crowding and isolation to determine whether serotonin in the brain regulates phase change. In addition, we cloned serotonin receptors and studied the expression of these receptors during phase change to determine whether serotonin receptors mediate behavioral phase change in the migratory locust. The results showed that serotonin levels in brains of gregarious nymphs significantly increased during isolation, and injection of serotonin into the brain accelerated the behavioral transition from the gregarious to solitary phase in isolated gregarious individuals. Although we found that the expression pattern of the *5*-*HT*_*2*_ receptor subtype increased in the brain during crowding, activating serotonin-5-HT_2_ signaling with a pharmaceutical agonist inhibited the gregarious behavior of solitary locusts. In addition, inactivation of this signaling via RNA interference (RNAi) had no effect on the gregariousness of locusts.

## Materials and methods

### Animals

The migratory locusts used in this study were from colonies maintained in the Institute of Zoology, Chinese Academy of Sciences, Beijing, China. Gregarious nymphs were cultured in large boxes (40 × 40 × 40 cm^3^) at a density of 500–1000 insects per container. Solitary nymphs were obtained from the gregarious colony and cultured alone in white metal boxes (10 × 10 × 25 cm^3^) supplied with charcoal-filtered compressed air for at least three generations before experimentation. The gregarious and solitary colonies were maintained under a 14 h light/10 h dark cycle at 30 ± 2°C and fed on fresh wheat seedlings and bran (Kang et al., 2004).

### High-performance liquid chromatography (HPLC) with electrochemical detection (ECD)

Serotonin in the brains (without optic lobe) of the migratory locust was quantified with reverse-phase high performance liquid chromatography (HPLC) with electrochemical detection (ECD). The brain tissues of the fourth-stadium solitary, gregarious, crowded, and isolated nymphs were immediately dissected and stored in liquid nitrogen. Ten brains per sample were homogenized using a mortar and pestle pre-cold with liquid nitrogen. Pulverized brain tissue was transferred to 1.5 ml Eppendorf tubes (Eppendorf International, Hamburg, Germany), and then lysed in 400 μ L ice-cold 0.1 M perchloric acid (Sigma–Aldrich) on ice for 10 min. The homogenates were centrifuged at 14,000× *g* for 10 min at 4°C. The supernatants were passed through 0.45 μm filters (Millipore Corporation, Billerica, MA, USA), transferred to new Eppendorf tubes, and stored at −80°C until HPLC-ECD analysis. Forty μ L supernatants were automatically loaded onto a quaternary low-pressure pump (Waters Corporation, e2695, Milford, MA, USA) with a C18 reverse phase column (Atalantis™ dC18, 2.1 × 150 mm, 3 μm, Waters Corporation). The electrode potential in the electrochemical detector was set at 800 mV. The mobile phase (pH 3.00) was composed of 7% acetonitrile (J&K Scientific Ltd., Beijing, China), 90 mM monobasic phosphate sodium (Sigma–Aldrich), 50 mM citric acid (Sigma–Aldrich), 2 mM octanesulfonic acid (J&K Scientific Ltd., Beijing, China), 2 mM NaCl (Sigma–Aldrich), and 50 μM EDTA (Sigma–Aldrich). The flow rate was adjusted to 0.25 mL min^−1^, and the temperature was set at 35°C. Data analysis was performed using Empower software (Waters Corporation). The serotonin levels were quantified by referring to external standards. The standard curve was generated with serial dilutions of standard solution containing serotonin (Sigma-Aldrich).

### Phylogenetic analysis of serotonin receptors

The sequences of serotonin receptors in the migratory locust were cloned by referring to putative sequences in the whole-genome database of *Locusta migratoria*. The other amino-acid sequences of serotonin receptors used for phylogenetic analysis were identified in the NCBI databases. To classify the subtypes of serotonin receptors, multiple sequence alignments of these insect serotonin receptors were performed with Clustal W and curated in MEGA 5.34 (Tamura et al., 2011). Neighbor-joining analysis was performed in MEGA 5 with bootstrapping 1000 replicates. GenBank accession numbers of protein sequences used were as follows: *Antheraea* 5-HT_1A_, ABY85410; *Antheraea* 5-HT_1B_, ABY85411; *Apis* 5-HT_1_, CBI75449; *Apis* 5-HT_2β_, CBX90121; *Apis* 5-HT_2α_, CBX90120; *Apis* 5-HT_7_, CAJ28210; *Bombyx* 5-HT, NP_001037502; *Danaus* 5-HT, EHJ69998; *Danaus* 5-HT, EHJ73524; *Drosophila* 5-HT_1A_, NP_476802; *Drosophila* 5-HT_1B_, NP_523789; *Drosophila* 5-HT_7_, NP_524599; *Drosophila* 5-HT_2β_, NP_649806; *Drosophila* 5-HT_2B_, NP_730859; *Gryllus* 5-HT_7_, BAJ83482; *Gryllus* 5-HT_1B_, BAJ83480; *Gryllus* 5-HT_1A_, BAJ83479; *Gryllus* 5-HT_2α_, BAJ83481; *Heliothis* 5-HT, CAA64863; *Manduca* 5-HT_1A_, ABI33826; *Manduca* 5-HT_1B_, ABI33827; *Papilio* 5-HT, BAD72868; *Periplaneta* 5-HT_1_, CAX65666; *Tribolium* 5-HT_7_, XP_966577; *Tribolium* 5-HT, XP_967449; *Tribolium* 5-HT_2*a*_, XP_972327.

## Experimental samples

### Isolation of gregarious locusts

The fourth-stadium gregarious nymphs were isolated and individually reared as solitary nymphs. After 1, 4, 16, or 32 h of isolation, the brains of isolated nymphs were dissected and immediately placed in RNAlater Solution (Ambion, Austin, Texas, USA) for qRT-PCR analysis. The brains of gregarious nymphs maintained in groups were sampled as the control group. To avoid the effects of circadian rhythm on phenotype of gregarious nymphs, all insects were sampled at the same time point for four biological replicates, and equal numbers of male and female insects were sampled per biological replicate.

### Crowding of solitary locusts

Ten solitary nymphs at the fourth stadium were introduced into an optic perplex-made box (10 × 10 × 10 cm^3^) and allowed to live with the gregarious nymphs at the same stadium. After staying with the stimulus group and crowding for 1, 4, 16, or 32 h, the brains of the crowded nymphs were dissected and immediately placed in RNAlater Solution for subsequent qRT-PCR analysis. The brains of solitary nymphs were sampled as the control group. All insects were sampled at the same time point for eight biological replicates, and equal numbers of male and female insects were sampled per biological replicate.

### RNA preparation

To eliminate the heterogeneity of different samples, total RNA was extracted from the brain tissues of controlled and treated nymphs. The brains were transferred into the lysis buffer and total RNA was extracted following the protocol of an RNAeasy mini kit (QIAGEN, Hilden, Germany).

### qRT-PCR

For analysis of the transcript of the three receptors, 2 μ g total RNA was reverse-transcribed using MMLV reverse transcriptase (Promega, Madison, USA) following the manufacturer's instructions. PCR reactions were performed in a 20 μ l volume and the final concentration of the primers was 250 nM. PCR amplification was conducted using RealMaster-Mix (SYBR Green) kit (Tiangen, Beijing, China), initiated with a 2 min incubation at 95°C, followed by 40 cycles of 20 s at 95°C, 20 s at 58°C and 20 s at 68°C. Four to eight biological replicates were performed for each sample. The expression of *5*-*HT*_*1*_, *5*-*HT*_*2*_, *5*-*HT*_*7*_ and the housekeeping gene ribosomal protein 49 (*RP49*) were detected using a Roche LightCycler 480. The standard curves for target genes (*5*-*HT*_*1*_, *5*-*HT*_*2*_, *5*-*HT*_*7*_) and reference genes (*RP49*) were generated with serial (10×) dilutions of plasmids. Efficiency of qRT-PCR and correlation coefficients were determined for the primers of each gene. We normalized all relative expression levels of three genes against the reference gene. Melting curve analysis was performed to confirm the specificity of amplification and all amplification products revealed a single melting peak. All PCR products were sequenced to confirm their identity before qRT-PCR experiments. The primers for qRT-PCR are listed in Table 1.

**Table 1:** **Primer sequences for qRT–PCR**.

**Genes**	**Forward primer (5′–3′)**	**Reverse primer (5′–3′)**
*5*-*HT*_*1*_	TGGGCAACGAGCACGAGGA	GCTCGTTGCCCAGGATGAG
*5*-*HT*_*2*_	CCGCGTCACGCTCAAGATC	AGGCTCATGGCGATGGAGA
*5*-*HT*_*7*_	AGTGCCAGGTGTGCCAGA	GTCGCCCTACATCTTCCT
*RP49*	CGCTACAAGAAGCTTAAGAGGTCAT	CCTACGGCGCACTCTGTTG

### Behavioral pharmacology in gregarious nymphs

To clearly determine the role of serotonin in regulating phase change of locusts, we chose the central position between the paired compound eyes to inject serotonin (5 mM × 3 μ L; Sigma–Aldrich) into the anterior of the locust brain using a micro-syringe with a depth of 1–2 mm. We made sure the tip of the micro-syringe was injected from ventral to dorsal direction of the locust head to avoid damaging the brain. The fourth-stadium gregarious nymphs injected with serotonin were immediately isolated for 15 or 30 min before the behavior assay. The control group received the same volume of saline for the behavioral assay.

Ketanserin and methiothepin (Sigma–Aldrich), antagonists of serotonin receptors, were applied as a cocktail to cover the range of possible pharmacological types of serotonin receptors in locusts as described in detail by Anstey et al. (2009). The antagonist cocktail (5 mM × 3 μ L) was injected into the head cavities of fourth-stadium gregarious nymphs in the same manner as serotonin. Behavior was assayed 15 or 30 min after isolation. The control group received the same volume of saline for the behavioral assay.

### Behavioral pharmacology in solitary nymphs

To analyze the role of 5-HT_2_ in crowding of the migratory locust, (±)-1-(2,5-Dimethoxy-4-iodophenyl)-2-aminopropane hydrochloride [(±)-DOI] (5 mM × 70 nL; Sigma–Aldrich), a 5-HT_2_ agonist (Colas et al., 1995; Johnson et al., 2009), was injected into the brains of fourth-stadium solitary nymphs 1 h before the behavioral assay. Briefly, the fourth-stadium nymphs were placed in a Kopf stereotaxic frame specially adapted for locust surgery. A midline incision was cut in the central position between the paired compound eyes using Nevis scissors to expose the underlying brain. All injections were performed under anatomical lens using a NANOLITER injector 2000 (World Precision Instruments, Sarasota, FL, USA) with a glass micropipette tip. The fourth-stadium solitary nymphs were put back into the solitary-rearing cages or were introduced into an optic perplex-made box (10 × 10 × 10 cm^3^) and allowed to live with the gregarious nymphs at the same stadium for 1, 4, 16, or 32 h. Again, all control groups received the same volume of saline for the behavioral assay.

### RNAi and behavioral assay in solitary nymphs

Double-strand RNA (dsRNA) of green fluorescent protein (GFP) and *5*-*HT*_*2*_ were prepared using the T7 RiboMAX Express RNAi system (Promega, Madison, USA) according to the manufacturer's instructions. The primers for dsRNA preparation are listed in Table 2. To find the optimal amount of dsRNA for RNAi injection and behavioral assay, we directly injected 12, 24, or 36 ng of dsRNA into the brains of fourth-stadium solitary nymphs as described above. After dsRNA injection, the solitary nymphs lived alone in solitary-rearing cages, and the effects of RNAi on the relative mRNA level were detected by qRT-PCR after 72 h. After then, the dsRNA-injected solitary nymphs were directly assayed or moved to the environment with gregarious nymphs (10 × 10 × 10 cm^3^) and crowded for 1, 4, 16, or 32 h, respectively, before behavioral assay.

**Table 2:** **Primer sequences for RNAi**.

**Genes**	**Forward primer (5′–3′)**	**Reverse primer (5′–3′)**
*5*-*HT*_*2*_	CTTCTTCGTGCTCAACCTG	GGAATGTATGAGGTCGTGAG
*GFP*	CACAAGTTCAGCGTGTCCG	GTTCACCTTGATGCCGTTC

### Behavioral assay in arena

We used the EthoVision system (Noldus Inc. Wageningen, the Netherlands) for video recording and data extraction. The arena behavior assay was performed in a rectangular arena (40 × 30 × 10 cm^3^). The wall of the arena is opaque plastic and the top is clear. One of the separated chambers (7.5 × 30 × 10 cm^3^) contained 20 fourth-stadium gregarious locusts as the stimulus group, and the other end of the chamber with the same dimensions is left empty. Both ends of the chamber were illuminated equally to prevent the formation of mirror images. The floor of the open arena was covered with filter paper during the behavior assay. The locust nymphs were gently transferred by a tunnel to the arena. Every insect was recorded for 6 min and examined only once (Roessingh et al., 1993; Anstey et al., 2009; Guo et al., 2011; Ma et al., 2011).

To measure and evaluate the phase state of the fourth-stadium solitary and gregarious nymphs, we constructed a binary logistic regression model in SPSS 15.0 to measure and quantify their behavioral phenotype (Ma et al., 2011). Eleven different behavioral parameters were expressed as a mixture of behavioral or categorical markers acquired as follows: entry frequency in the stimulus area (EFISA, stimulus area was defined as 25% of the arena closest to the stimulus group), latency of first occurrence in stimulus area (LFOISA), total duration in the area close to the arena wall (TDCW), entry frequency in the area close to the arena wall (EFCW), entry frequency in the region opposite the stimulus area (EFIOSA, the opposite of the stimulus area was defined as 25% of the arena at the opposite end of the stimulus group), latency of first occurrence opposite the stimulus area (LFOIOSA), mean distance to the stimulus group (MDTSG), total distance moved (TDM), total duration of movement (TDMV) as well as frequency of movement (FOM) and attraction index (AI, AI stands for the extent of tested animals attracted by the stimulus group. AI = total duration in stimulus area—total duration in opposite area). In total we measured 100 solitary nymphs and 100 gregarious nymphs at the fourth stadium for extraction of behavioral data. A forward stepwise approach was applied to build the logistic regression model by using the untransformed data of these 11 behavioral parameters. The building process was finished when no more improvement occurred by adding more behavior variables. The independent variables in the logistic regression model were closely correlated with the significant level of their regression coefficient β in the Wald test. The behavioral parameters of this model were adjusted until the regression model discriminated the two phases at the optimum level according to the following equation: P-sol = eη/(1 + eη), where η = β0 + β1 · X1 + β2 · X2 +…+ βk · Xk, X1, X2, …, Xk are the behavioral covariates. P-sol is the probability that the nymphs should be regarded as a member of the solitary phase population, with a value ranging from 1 to 0, where 1 means that individuals display solitary behavior and 0 indicates that individuals display gregarious behavior (Table 3). The most robust indicators of the phase state were retained in the model. This model shares similar features with previous regression models used for binary discrimination of solitary and gregarious locusts (Roessingh et al., 1993; Anstey et al., 2009; Guo et al., 2011; Ma et al., 2011).

**Table 3:** **Behavioral variables retained in the best-fitting logistic regression model obtained from the fourth-stadium solitary and gregarious nymphs**.

**Parameters in equation**	**β**	***SE***	**Wald**	***df***	**Sig.**	**Exp(β)**
TDM	−0.016	0.004	14.286	1	0.000	0.984
FOM	−0.172	0.073	5.494	1	0.019	0.842
AI	−0.005	0.001	20.571	1	0.000	0.995
Constant	2.361	0.353	44.773	1	0.000	10.600

This table indicates the significance levels of the individual model parameter for the behavioral discrimination of the locust nymphs. Abbreviations: TDM, total distance moved; FOM, frequency of movement; AI, attraction index.

### Statistical analysis

Expression levels of serotonin receptors between gregarious and solitary locusts were analyzed by the Student's *t*-test. Serotonin content and expression levels of serotonin receptors over a time course of crowding and isolation were analyzed by one-way analysis of variance (ANOVA). The serotonin content and expression levels of receptors were expressed as the mean ± standard error of the mean (SEM). Behavioral data were analyzed by the Mann-Whitney *U*-test because of the non-normal distribution characteristics. Differences were considered significant at *p* < 0.05. The probabilistic metric of solitariness (P-sol) is presented as the median value. All the statistics were analyzed using SPSS 15.0 (SPSS Inc., Chicago, IL, USA).

## Results

### Changes in serotonin content during the phase change of locusts

**Figure 1:**
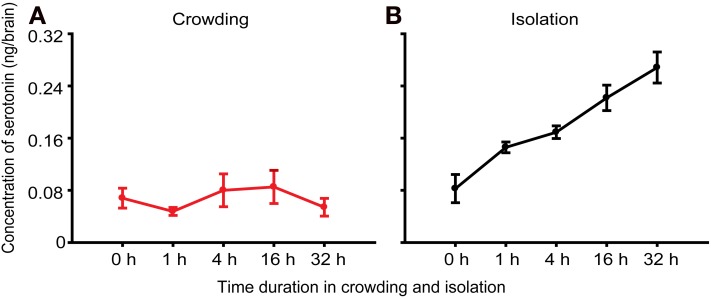
**Concentration of serotonin in brains of the migratory locust. (A)** Concentration of serotonin in the brains of solitary nymphs subjected to crowding [One-way ANOVA, *F*_(4, 35)_ = 0.529, *P* = 0.753]. **(B)** Concentration of serotonin in the brains of gregarious nymphs subjected to isolation [One-way ANOVA, *F*_(4, 35)_ = 40.890, *P* < 0.001]. The data represent mean values and error bars represent SEM.

To confirm whether serotonin regulated behavioral differences between solitary and gregarious phase, we measured serotonin levels in the brains of solitary and gregarious nymphs and found no difference between the two phases of the migratory locust (Ma et al., 2011). To explore the role of serotonin in the phase change of the locusts, we first analyzed the fluctuation of serotonin levels over a time course of crowding and isolation to examine the causal relationship of serotonin with phase change. Results revealed that the serotonin level in the brain did not change during the crowding of the solitary nymphs, but the level of serotonin increased during the isolation of gregarious nymphs (Figures 1A,B). These results indicate that changes in serotonin levels in the brain are associated with the solitariness of the gregarious nymphs.

### Amino acid sequence alignment and a phylogenetic analysis of serotonin receptors

**Figure 2:**
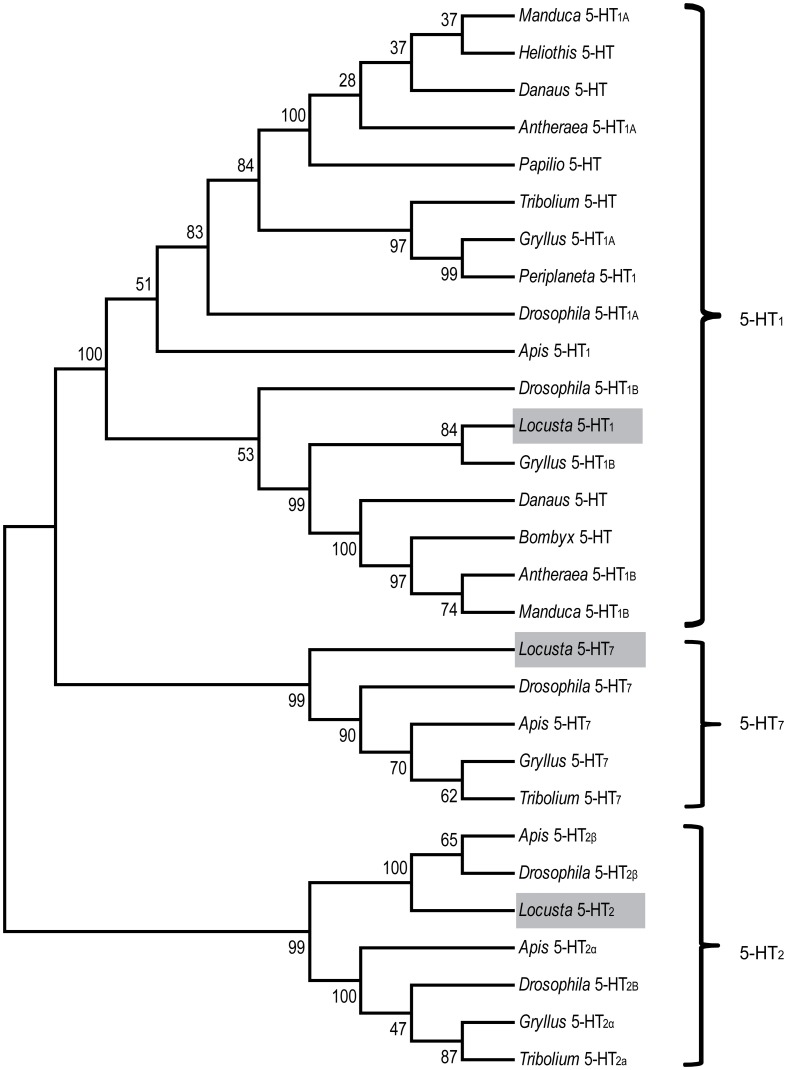
**Phylogenetic tree comparing insect serotonin receptors.** Phylogenetic and molecular evolutionary analyses were conducted using MEGA 5. The serotonin receptors of the migratory locust (*Locusta* 5-HT_1_, 5-HT_2_, and 5-HT_7_) are indicated by gray bars. Abbreviations: *Antheraea, Antheraea pernyi; Apis, Apis mellifera; Danaus, Danaus plexippus; Drosophila, Drosophila melanogaster; Gryllus, Gryllus bimaculatus; Heliothis, Heliothis virescens; Manduca, Manduca sexta; Bombyx, Bombyx mori; Locusta, Locusta migratoria; Papilio, papilio xuthus; Periplaneta, Periplaneta americana; Tribolium, Tribolium castaneum*.

**Figure 3:**
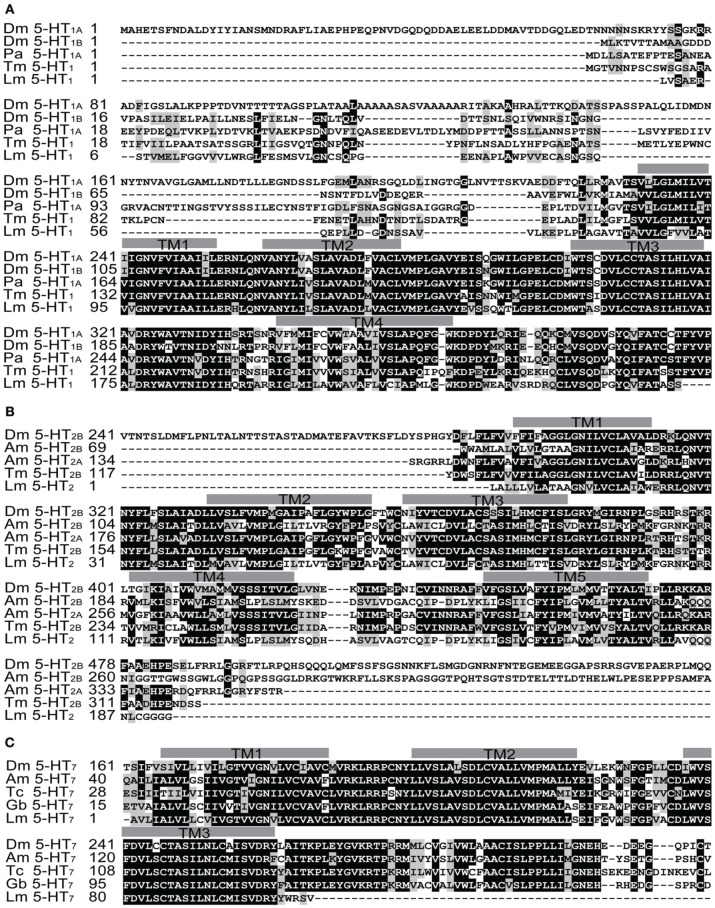
**Amino acid sequence alignment of serotonin receptors in the migratory locust and the ones in other insects. (A)** Alignment of the amino acid sequences of Lm 5-HT_1_ with orthologous receptors from *Drosophila melanogaster* (Dm 5-HT_1A_, NP_476802; Dm 5-HT_1B_, NP_523789), *Periplaneta americana* (Pa 5-HT_1A_, CAX65666), and *Tribolium castaneum* (Tm 5-HT_1_, XP_967449). **(B)** Sequence alignment of Lm 5-HT_2_ with orthologous receptors from *Drosophila melanogaster* (Dm 5-HT_2B_, NP_730859), *Apis mellifera* (Am 5-HT_2A_, CBX90120; Am 5-HT_2B_, CBX90121), and *Tribolium castaneum* (Tc 5-HT_2B_, NP_972327). **(C)** Sequence alignment of the Lm 5-HT_7_ with orthologous receptors from *Drosophila melanogaster* (Dm 5-HT_7_, NP_524599), *Apis mellifera* (Am 5-HT_7_, CAJ28210), *Tribolium castaneum* (Tc 5-HT_7_, XP_966577), and *Gryllus bimaculatus* (Gb 5-HT_7_, BAJ83482). Identical residues of the aligned sequences are shown as white letters against black ones, whereas conservatively substituted residues are shaded. Putative transmembrane regions are indicated by gray bars. Dashes indicate gaps that were introduced to maximize homology.

G protein-coupled receptor pathways regulated by neurotransmitters and neuromodulators have been suggested to regulate the phase change of locusts (Chen et al., 2010). As a ligand of G protein-coupled receptors, serotonin controls diverse behaviors in animals (Nichols and Nichols, 2008; Berg and Clarke, 2009). To clone serotonin receptors and discover their roles in phase change, we first collected the putative receptor gene sequences in the whole-genome database of *Locusta migratoria* using a BLAST search. Referring to these putative sequences, we cloned the partial sequences of three orthologous genes (*5*-*HT*_*1*_, *5*-*HT*_*2*_, and *5*-*HT*_*7*_) from the locust brain, and the cDNA fragment length of these three receptors were 753, 585, and 315 bp, respectively. To validate and classify the subtypes of serotonin receptors, we performed a phylogenetic analysis using MEGA5.34 (Tamura et al., 2011). The result showed that the three orthologous receptors belonged to the three insect serotonin receptors families (Figure 2). The conserved transmembrane (TM) segments of serotonin receptors were analyzed by the TMHMM Server 2.0 (Sonnhammer et al., 1998). The partial fragment of 5-HT_1_ receptors encoded four TM segments that corresponded to the first four GPCR TM segments (TM1–TM4 in Figure 3A), whereas the fragment of 5-HT_2_ receptors included the five TM segments that corresponded to the first to the fifth GPCR TM segments (TM1–TM5 in Figure 3B). Moreover, the three TM regions encoded by the partial sequence of 5-HT_7_ were the first to third GPCR TM segments (TM1–TM3 in Figure 3C).

### Expression patterns of serotonin receptors during the phase change of locusts

**Figure 4:**
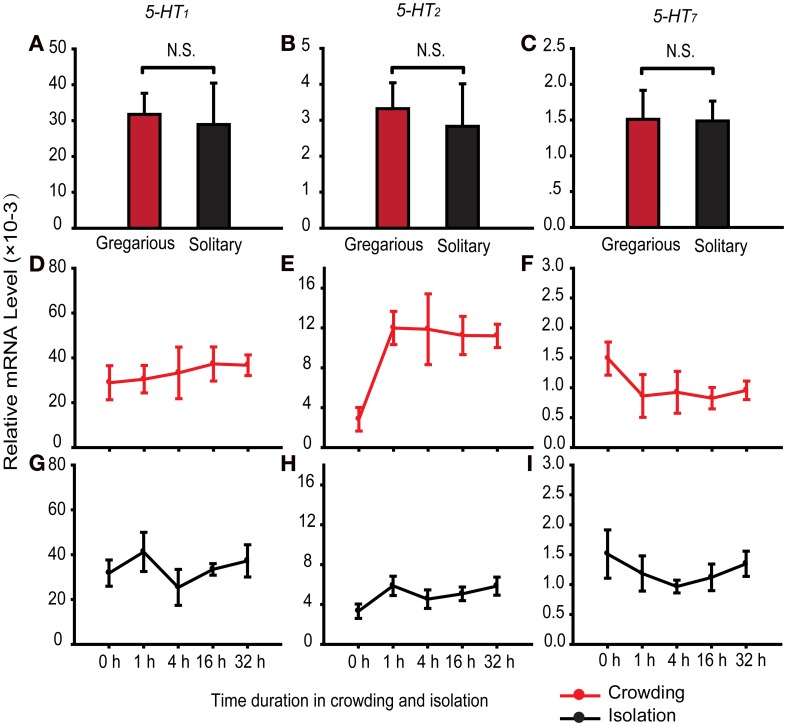
**Expression patterns of serotonin receptors in migratory locust brains. (A–C)** Expression patterns of *5-HT_1_* **(A)**, *5-HT_2_* **(B)**, and *5-HT_7_* **(C)** in the brains of gregarious and solitary nymphs [Student's *t*-test, *t*_(0.05/2, 14)_ = 0.22, *P* = 0.833 for *5-HT_1_*; *t*_(0.05/2, 14)_ = 0.35, *P* = 0.736 for *5-HT_2_*; *t*_(0.05/2, 14)_ = 0.05, *P* = 0.962 for *5-HT_7_*]. **(D–F)** Expression patterns of *5-HT_1_* **(D)**, *5-HT_2_* **(E)**, and *5-HT_7_* **(F)** in the brains of fourth-stadium solitary nymphs during crowding [One-Way ANOVA, *F*_(4, 35)_ = 0.245, *P* = 0.937 for *5-HT_1_*; *F*_(4, 35)_ = 4.574, *P* = 0.003 for *5-HT_2_*; *F*_(4, 35)_ = 1.756, *P* = 0.173 for *5-HT_7_*]. **(G–I)** Expression patterns of *5-HT_1_* **(G)**, *5-HT_2_* **(H)**, and *5-HT_7_* **(I)** in the brains of fourth-stadium gregarious nymphs during isolation [One-Way ANOVA, *F*_(4, 15)_ = 1.240, *P* = 0.332 for *5-HT_1_*; *F*_(4, 15)_ = 1.163, *P* = 0.207 for *5-HT_2_*; *F*_(4, 15)_ = 1.824, *P* = 0.159 for *5-HT_7_*]. The data represent mean values and error bars represent SEM. Abbreviations: G, gregarious; S, solitary. N.S., not significant.

To determine the role of serotonin receptors in regulating behavioral differences between two phases, we detected the expression of the three serotonin receptors *5-HT_1_*, *5-HT_2_*, and *5-HT_7_* in the brains of solitary and gregarious individuals. The three receptors did not show differential expression in fourth-stadium solitary and gregarious nymphs (Figures 4A–C).

To determine the causal relationship of serotonin receptors in regulating behavioral phase change, we detected the expression patterns of the three serotonin receptors during phase change of locusts. In the time course of crowding in solitary nymphs, we found that *5-HT_2_* was up-regulated after 1 h of crowding and remained stable thereafter (Figure 4E). The receptors *5-HT_1_* and *5-HT_7_* were not differentially expressed over the time course of crowding (Figures 4D,F). In addition, the expression level of the three receptors did not change over the time course of isolation (Figures 4G–I). Thus, *5-HT_2_* may respond to the gregariousness of solitary nymphs.

### Role of serotonin in the isolation of gregarious nymphs

**Figure 5:**
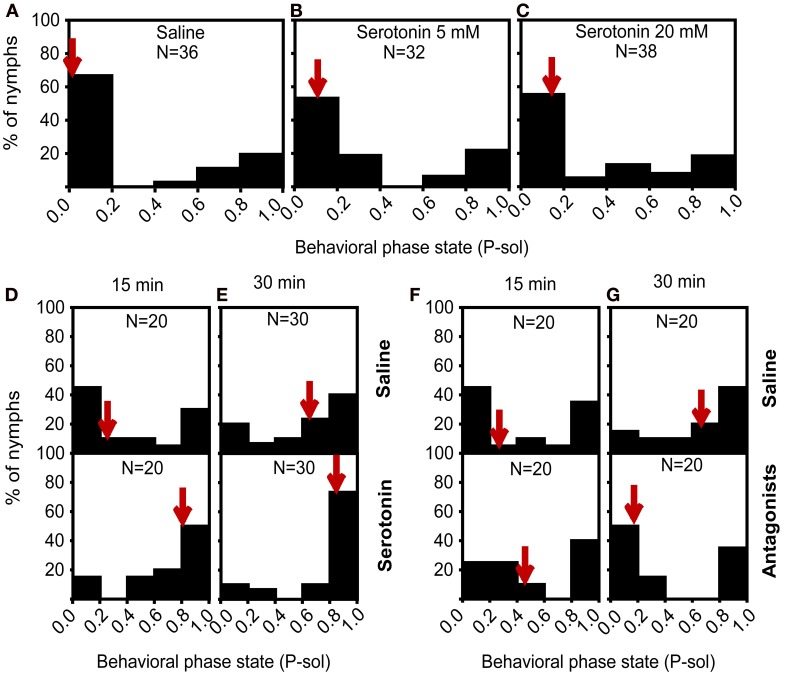
**Behavioral responses of gregarious nymphs after pharmacological intervention in serotonin signaling. (A–C)** Effect of serotonin on the phase state of gregarious nymphs, saline **(A)**, 5 mM **(B)**, and 20 mM **(C)**. **(D,E)** Effect of serotonin on the phase state of gregarious nymphs isolated for 15 min **(D)** and 30 min **(E)**. **(F,G)** Effects of serotonin receptor antagonists on the phase state of gregarious nymphs isolated for 15 min **(F)** and 30 min **(G)**. P-sol is probabilistic metric of solitariness. Arrows indicate median P-sol values.

Given the increase of the serotonin levels with behavioral changes over the time course of isolation, we hypothesized that serotonin played a role in the solitariness of gregarious nymphs. To validate this assumption, we injected different doses of serotonin into the head cavities of fourth-stadium gregarious nymphs to optimize serotonin concentration. Behavioral assay revealed that 5 mM and 20 mM serotonin did not affect the behavioral state of gregarious nymphs (Mann–Whitney *U*-test, *U* = 468, *P* = 0.172 for 5 mM; *U* = 609, *P* = 0.404 for 20 mM) (Figures 5A–C). Subsequently, we injected 5 mM serotonin into the gregarious nymphs and then exposed them to 15 or 30 min of isolation before behavioral assay. We found that the gregarious nymphs injected with serotonin followed by 15 min of isolation showed a significant behavioral shift toward the solitary phase state, with 50% of injected nymphs at P-sol interval of 0.8–1.0, compared with the control group (Mann–Whitney *U*-test, *U* = 117, *P* = 0.024) (Figure 5D). Serotonin administration with 30 min of isolation also resulted in a behavioral shift from the gregarious to solitary state, with 73.3% of injected solitary nymphs at P-sol interval = 0.8–1.0 compared with the controls subjected to 30 min of isolation (Mann–Whitney *U*-test, *U* = 310, *P* = 0.038) (Figure 5E). Results confirmed the hypothesis that serotonin regulated the solitariness of gregarious locusts.

We inactivated serotonin receptors through pharmacological intervention to further verify whether serotonin mediated the isolation of gregarious nymphs. Injection of a antagonist cocktail (ketanserin and methiothepin) coupled with 15 min of isolation did not affect the behavior changes (Mann–Whitney *U*-test, *U* = 172, *P* = 0.461) (Figure 5F). However, the locusts treated with the antagonist cocktail in the head cavity significantly shifted their behavior from the solitary to gregarious phase after 30 min of isolation, with 50% of the injected ones at P-sol interval = 0.0–0.2 (Mann–Whitney *U*-test, *U* = 110, *P* = 0.014) (Figure 5G). Antagonist-injected gregarious nymphs exposed to isolation for 30 min showed full gregarious behavior. These results indicate that interfering with serotonin receptors during isolation prevents locust solitariness.

### Role of the serotonin receptor in the crowding of solitary nymphs

**Figure 6:**
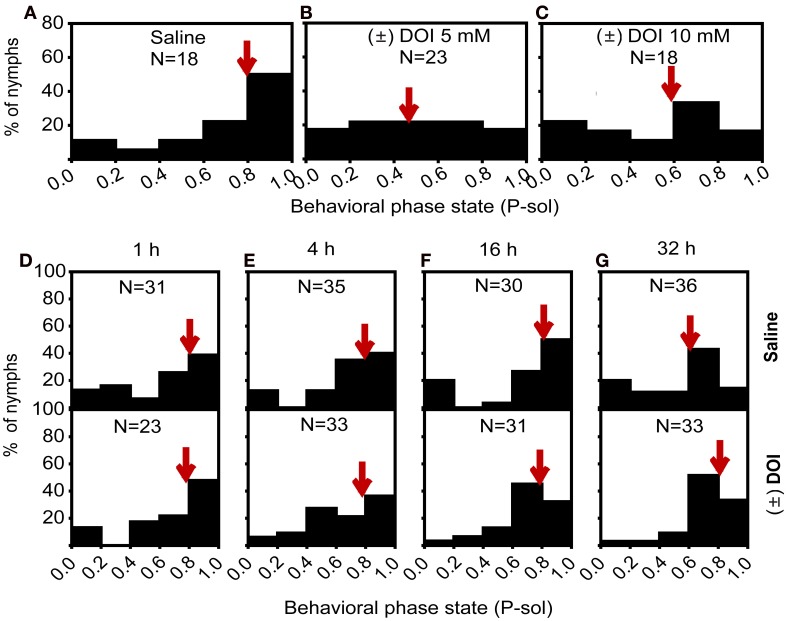
**Behavioral responses of solitary nymphs after activating the 5-HT_2_ signaling pathway. (A–C)** Effect of (±) DOI on the phase state of solitary nymphs, saline **(A)**, 5 mM **(B)**, and 10 mM **(C)**. **(D–G)** Effect of (±) DOI on the phase state of solitary nymphs subjected to crowding for 1 h **(D)**, 4 h **(E)**, 16 h **(F)**, and 32 h **(G)**. P-sol is probabilistic metric of solitariness. Arrows indicate median P-sol values.

Increased *5-HT_2_* expression in the brain of locusts suggested the role of this receptor in regulating the gregariousness of locusts. To further clarify the role of serotonin receptor in regulating the gregariousness of solitary nymphs, we injected the serotonin receptor 5-HT_2_ agonist (±)-DOI into the fourth-stadium solitary nymphs and detected the function of serotonin-5-HT_2_ signaling during time course crowding. We injected different (±)-DOI doses into the brain and examined the behavioral responses. Results showed that the solitary nymphs injected with (±)-DOI (5 mM and 10 mM) shifted toward the gregarious phase in terms of behavior traits (Mann–Whitney *U*-test, *U* = 199, *P* = 0.001 for 5 mM; *U* = 94, *P* = 0.031 for 10 mM) (Figures 6A–C). However, the 5 mM (±)-DOI-injected solitary nymphs still showed solitary behavior during the time course of crowding, even after crowding 32 h the control group displayed significant behavioral change (Mann–Whitney *U*-test, *U* = 146, *P* = 0.442 for 1 h; *U* = 647, *P* = 0.868 for 4 h; *U* = 146, *P* = 0.103 for 16 h; *U* = 334, *P* = 0.002 for 32 h) (Figures 6D–G). Consistent with the previous study that the solitary nymphs displayed significant behavioral changes after crowding 32 h (Guo et al., 2011), (±)-DOI injection inhibited this behavioral shift compared to the control group (Figure 6G). Therefore, activation of 5-HT_2_ signaling inhibited gregariousness during the crowding process.

**Figure 7:**
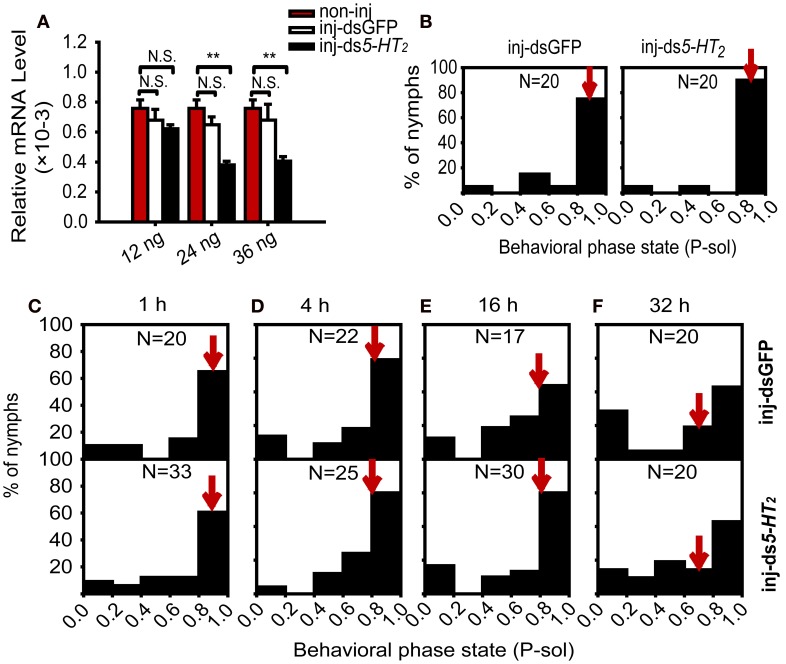
**Behavioral responses of solitary nymphs after RNAi knockdown of *5-HT_2_*. (A)** Effects of RNAi on the relative mRNA level [Student's *t*-test, *t*_(0.05/2, 14)_ = 1.656, *P* = 0.129 for 12 ng; *t*_(0.05/2, 14)_ = 6.226, *P* < 0.001 for 24 ng; *t*_(0.05/2, 14)_ = 5.553, *P* < 0.001 for 36 ng]. In all groups, treatment (inj-GFP, inj-ds*5-HT_2_*) compared with the corresponding non-injected controls. The data represent mean values and error bars represent SEM. **(B)** Behavioral responses of solitary nymphs after RNAi knockdown of *5-HT_2_*. **(C–F)** Behavioral responses of solitary nymphs after RNAi knockdown of *5-HT_2_* with crowding for 1 h **(C)**, 4 h **(D)**, 16 h **(E)**, and 32 h **(F)**. P-sol is probabilistic metric of solitariness. Arrows indicate median P-sol values. ^**^P < 0.01; N.S., not significant.

To further explore the role of 5-HT_2_ signaling in crowding of solitary nymphs, we analyzed the effect of *5-HT_2_* RNAi knockdown on the behavioral state of solitary nymphs exposed to crowding. First, we tested the efficiency of RNAi knockdown using different doses of double-stranded RNA. Results showed that the 5-HT_2_ mRNA level significantly decreased after injecting 24 or 36 ng of dsRNA, but no change was detected in the dsGFP-injected controls and 12 ng ds*5-HT_2_* groups (Figure 7A). In the solitary nymphs, *5-HT_2_* RNAi knockdown did not affect the behavioral state (Mann–Whitney *U*-test, *U* = 146, *P* = 0.149) (Figure 7B). In addition, *5-HT_2_* RNAi knockdown did not influence the behavioral state and behavioral shift of solitary nymphs compared with dsGFP-injected groups during the crowding process (Mann–Whitney *U*-test, *U* = 322, *P* = 0.883 for 1 h; *U* = 264, *P* = 0.823 for 4 h; *U* = 237, *P* = 0.689 for 16 h; *U* = 181, *P* = 0.327 for 32 h) (Figures 7C–F).

## Discussion

In this study, during the isolation of the gregarious nymphs, the concentration of serotonin increased significantly, whereas serotonin receptors expressed in a stable manner. The injection of serotonin in the isolated nymphs promoted the behavioral change from gregarious to solitary phase. During the crowding of solitary nymphs, the serotonin level in the brain remained unchanged, whereas *5-HT_2_* increased after 1 h of crowding and showed stable expression level thereafter. Activation of 5-HT_2_ inhibited the gregariousness of solitary nymphs in crowding treatment. Thus, serotonin promoted the phase change from gregarious to solitary phase.

### Serotonin accelerates the behavioral change from gregarious to solitary phase during isolation

In this study, we verified that injection of serotonin in the brain did not induce solitariness in gregarious nymphs, but injection of serotonin did accelerate the behavioral transition to the solitary phase during isolation of gregarious nymphs. Blocking the function of serotonin by injection of serotonin receptor antagonist cocktails prevented the behavioral shift from the gregarious to solitary phase during isolation. These results suggest that increase of serotonin levels during isolation are a result of the process of gregarious nymph isolation. In addition, the increase in serotonin levels and the stable expression of serotonin receptors during isolation suggest that serotonin signaling mediated this process at the neurochemical level, but not through mechanisms underlying receptor expression. Consistent with our hypothesis, gregarious nymphs treated with serotonin and coupled with 30 min of isolation showed solitary behavior, whereas the antagonist cocktail inhibited this transition. In the isolation process, the gregarious nymphs displayed a behavioral shift from attraction to repulsion responses and their levels of movement were reduced to the level of solitary controls (Guo et al., 2011). The phase change of locusts relies on their decision to join or leave the other locusts (Uvarov, 1966; Burrows et al., 2011; Guo et al., 2011), and serotonin reportedly mediates this decision to withdraw in invertebrates and vertebrates. In crickets, serotonin has been proposed to integrate agonistic signals for the decision to flee (Stevenson and Rillich, 2012). Similarly, serotonin limits impulsivity (Nelson and Trainor, 2007) or stimulates the drive to withdraw in mammals (Tops et al., 2009). Spoont (Spoont, 1992) reviewed that the increase in serotonin decreased sensory reactivity and protected against overstimulation; thus, serotonin probably reduces the responses and activities of gregarious nymphs to extrinsic factors. In addition, the depletion of serotonin induces hyperactivity and enhances the startle responses of crickets (Stevenson et al., 2000, 2005), whereas higher doses of serotonin inhibits the locomotion behavior of the juvenile lobster *Homarus americanus* (Peeke et al., 2000). Therefore, serotonin may integrate the outer stimuli for the decision to withdraw and inhibit locomotion during the isolation of gregarious nymphs. The fact that serotonin did not affect the behavior of the gregarious phase suggested that other modulatory mechanisms also played important roles in the isolation process.

### Activation of serotonin-5-HT_2_ signaling inhibits the gregariousness of solitary nymphs in crowding treatment

In our previous study, we found that injection of the serotonin precursor 5-HTP coupled with long-term crowding inhibited the gregariousness (Ma et al., 2011). In this study, we only discovered a significant increase in *5-HT_2_* expression among the three serotonin receptors during crowding. Although activation of 5-HT_2_ in solitary nymphs partially induced gregarious-like behavior, activation of 5-HT_2_ during crowding inhibited the performance of gregarious behavior of solitary nymphs. These results showed that activation of 5-HT_2_ induced different modulatory effects on behavior of solitary phase locusts and their crowding process. Similar to these results, serotonin modulated crayfish lateral giant escape command neurons in two different ways. Low concentration of serotonin or high concentration of serotonin reaching gradually resulted in facilitatory effects on those neurons. However, high concentration of serotonin reaching rapidly resulted in inhibitory effect on those neurons (Teshiba et al., 2001). In addition, low doses of exogenous serotonin in juvenile lobsters did not affect motor behavior but higher doses inhibited this behavior (Peeke et al., 2000). Thus, in solitary nymphs with low *5-HT_2_* expression, 5-HT_2_ agonist activates 5-HT_2_ and facilitates its role in regulating behavioral change toward the gregarious phase. By contrast, with increased expression of *5-HT_2_* during crowding, the activation of serotonin signaling pathways inhibits the phase change of solitary nymphs. Serotonin-5-HT_2_ signaling probably regulates phase change through dual and opposing modulatory mechanisms.

Here we found that serotonin accelerated solitariness in the isolation of gregarious nymphs. Activation of *5-HT_2_* signaling inhibited gregariousness during the crowding of solitary nymphs. In addition, RNAi knockdown of *5-HT_2_* did not influence the behavioral state and behavioral shift during crowding. These results indicate that the fluctuations of serotonin content and *5-HT_2_* expression are results of locust phase change and may be regulated by a set of differentially expressed genes or pathways involved in phase change. A previous study (Guo et al., 2011) identified a large number of differentially expressed genes related with isolation and crowding of locusts. *De novo* analysis of the migratory locust transcriptome revealed that many neurotransmitter receptors, synthetases, and transporters are differentially expressed between the two phases of fourth-stadium nymphs (Chen et al., 2010). The causal pathways or effectors involved in regulating phase change may result in changes in serotonin content and *5-HT_2_* expression during the phase change of locusts.

### Controversial role of serotonin in regulating the phase change of migratory locusts and desert locusts

In this study, we verified that serotonin mediated solitariness and inhibited gregariousness in the phase transition of migratory locusts. This finding is contradictory to the report that serotonin initiates the swarming behavior of the desert locust (Anstey et al., 2009). The controversial role of serotonin in regulating phase change in locusts may be explained by the following explanations.

First, the role of serotonin in regulating the phase change of locusts is closely related to target tissues subjected to experimental intervention. In a study on desert locusts, the thoracic ganglia was the target for treatment with serotonin and its agonist (Anstey et al., 2009). In the present study, pharmacological intervention focused on the brain of migratory locusts. The brain of an insect integrates multisensory inputs and directs patterns of activity ascended by “lower” neural centers, such as the thoracic ganglia (Delcomyn, 1999; Schaefer and Ritzmann, 2001; Zill, 2010). Moreover, many innate behaviors such as locomotion, feeding and mating are controlled by body ganglia but not the brain (Wessnitzer and Webb, 2006). The central pattern generator in the thoracic ganglia of the desert locust has been identified to regulate locomotion without control by the brain (Berkowitz and Laurent, 1996). The enhancement of locomotion in the crowding of the desert locust may result from the auto-feedback loop of the central pattern generator in the thoracic ganglia of the locust. However, much more complex and important behaviors of insects are integrated through the brain (Wessnitzer and Webb, 2006). In this study, we found that serotonin regulated the solitariness of migratory locust through the intervention of serotonin signaling in the brain. In addition, the systematic application of serotonin in a recent study on desert locusts showed no influence on behavioral phase change (Tanaka and Nishide, 2012). This injection method may merge effects of serotonin in different tissues, such as thoracic ganglia and brain. Therefore, we speculated that serotonin may function via tissue-specific signaling to regulate locust phase change; however, exactly how serotonin in the brain regulates the isolation process needs further exploration. Serotonin reportedly mediates the avoidance of threat (Deakin, 2003) and the withdrawal from dangerous, averse, or highly stimulating environment (Tops et al., 2009). The increase of serotonin in the brain of the migratory locust may signal overstimulation of threats and facilitate withdrawal from the groups. Meanwhile, serotonin probably suppresses the processing of sensory input and/or interrupts the motor output to the thoracic ganglia. The behavioral shift from the gregarious to the solitary phase induced by serotonin in the brain is probably a certain adaptive response to extrinsic factors affecting the migratory locust.

Second, the duration of treatment probably resulted in contradictory effects of serotonin or agonist between the two species. In the desert locust, the short-term application of serotonin or injection of its precursor in the thoracic ganglia induced gregarious behavior (Anstey et al., 2009). In contrast, the long-term and systematic injection of serotonin had no influence on attraction/repulsion behavior, one of the important parameters indicating behavioral phase change (Tanaka and Nishide, 2012). In the migratory locust, the long-term and systematic injection of serotonin precursor inhibited the gregariousness of the migratory locust (Ma et al., 2011). In this study, although solitary nymphs injected with serotonin receptor agonist in the brain showed partial gregarious behavior, the injection of this agonist in the brain coupled with crowding inhibited gregariousness after 32 h of crowding. Thus, serotonin may have a short-term auto evoking effect on outer stimuli but has no stable driving effect in the crowding of locusts.

In addition, the different performances of the conserved pathway underlying the regulation of phase change may result from the species-specific traits of the two locust species. Some regulating pathways, despite being conserved across taxa, may show differentiation between closely related species. For instance, ants and bees have an inverse relationship between foraging expression and behavior. The expression of this gene is up-regulated in honeybee foragers and down-regulated in red harvester ant foragers (Ingram et al., 2005). The serotonin pathway, which is conserved in animals, modulates phase change of migratory locusts and desert locusts (Anstey et al., 2009) through different regulating patterns. Moreover, previous studies have shown that the speed of crowding is slower than that of the isolation process in the migratory locust (Guo et al., 2011), whereas in the desert locust, the speed of crowding is quicker than that of the isolation process (Pener and Simpson, 2009). Thus, conserved molecular and neural pathways probably regulate behavioral patterns of the two locust species in a species-specific manner. The mechanism that regulates behavioral patterns of the two locust species still need to be further investigated in the future.

### Conflict of interest statement

The authors declare that the research was conducted in the absence of any commercial or financial relationships that could be construed as a potential conflict of interest.
